# Hemisphere-Specific Functional Remodeling and Its Relevance to Tumor Malignancy of Cerebral Glioma Based on Resting-State Functional Network Analysis

**DOI:** 10.3389/fnins.2020.611075

**Published:** 2021-01-13

**Authors:** Siqi Cai, Zhifeng Shi, Chunxiang Jiang, Kai Wang, Liang Chen, Lin Ai, Lijuan Zhang

**Affiliations:** ^1^Paul. C. Lauterbur Research Centers for Biomedical Imaging, Shenzhen Institutes of Advanced Technology, Chinese Academy of Sciences, Shenzhen, China; ^2^University of Chinese Academy of Sciences, Beijing, China; ^3^Department of Neurosurgery, Huashan Hospital of Fudan University, Shanghai, China; ^4^Beijing Neurosurgical Institute, Beijing Tiantan Hospital, Capital Medical University, Beijing, China

**Keywords:** cerebral glioma, resting state functional MRI, functional connectivity, topological feature, multivariate logistic regression

## Abstract

Background: Functional remodeling may vary with tumor aggressiveness of glioma. Investigation of the functional remodeling is expected to provide scientific relevance of tumor characterization and disease management of glioma. In this study, we aimed to investigate the functional remodeling of the contralesional hemisphere and its utility in predicting the malignant grade of glioma at the individual level with multivariate logistic regression (MLR) analysis. Subjects and Methods: One hundred and twenty-six right-handed subjects with histologically confirmed cerebral glioma were included with 80 tumors located in the left hemisphere (LH) and 46 tumors located in the right hemisphere (RH). Resting-state functional networks of the contralesional hemisphere were constructed using the human brainnetome atlas based on resting-state fMRI data. Functional connectivity and topological features of functional networks were quantified. The performance of functional features in predicting the glioma grade was evaluated using area under (AUC) the receiver operating characteristic curve (ROC). The dataset was divided into training and validation datasets. Features with high AUC values in malignancy classification in the training dataset were determined as predictive features. An MLR model was constructed based on predictive features and its classification performance was evaluated on the training and validation datasets with 10-fold cross validation. Results: Predictive functional features showed apparent hemispheric specifications. MLR classification models constructed with age and predictive functional connectivity features (AUC of 0.853 ± 0.079 and 1.000 ± 0.000 for LH and RH group, respectively) and topological features (AUC of 0.788 ± 0.150 and 0.897 ± 0.165 for LH and RH group, respectively) achieved efficient performance in predicting the malignant grade of gliomas. Conclusion: Functional remodeling of the contralesional hemisphere was hemisphere-specific and highly predictive of the malignant grade of glioma. Network approach provides a novel pathway that may innovate glioma characterization and management at the individual level.

## Introduction

Cerebral glioma is the most frequently identified intracranial tumor in adults. As gliomas with different malignancy may progress in distinct proliferation kinetics (Tubiana, 1989; Louis et al., 2016), it is necessary to preoperatively estimate the biological aggressiveness of gliomas for the therapeutic formulation. With the burgeoning development of artificial intelligence and radiomics, a combination of high dimensional features derived from multimodal neuroimaging has achieved plausible accuracy in reflecting the biological aggressiveness of glioma (Tian et al., 2018; Yang et al., 2018). However, disadvantages of these analysis paradigms still remain. Texture feature analysis is usually lesion-oriented and needs boundary delineation. This may over- or underestimate the malignancy of glioma, as non-neoplastic components such as peritumoral reaction and intratumoral necrosis would complicate the feature extraction. In addition, the effect of glioma-induced functional remodeling and its relevance to the clinical profile were largely overlooked. Recent studies demonstrated that the interaction between glioma and active neurons promotes tumor growth and shapes the overall patient survival (Venkatesh et al., 2015, 2019). This interaction may trigger network plasticity that varies with tumor kinetics and the biological aggressiveness (Kong et al., 2016). Therefore, characterization of the network remodeling is promising to provide useful markers to signify the tumor malignancy and disease dynamic of glioma.

Connectivity-based analysis is an approach of choice for exploring the functional remodeling of cerebral gliomas at both local and global levels. Brain networks of default mode, language and hand-motor showed profoundly reduced functional integrity (Briganti et al., 2012; Mallela et al., 2016; Kocher et al., 2020), and altered inter- and intra- network connectivity in the contralesional hemisphere in subjects with advanced gliomas (De Baene et al., 2017; Fox and King, 2018; Yuan et al., 2020). These connectivity-based findings indicate that the growth of glioma triggers both intra- and inter-hemispheric functional remodeling. In addition, the optimization of glioma treatment targeting maximal tumor removal while maximally preserving the subject’s functional integrity remains ungrounded (Müller et al., 2019). Connectivity-based analysis would enable itemizing the ascription of an individual brain area to the pathological network configuration and its relevance to the malignant grade of glioma. Therefore, characterization of the network remodeling is promising to provide useful markers to signify the tumor malignancy of glioma and new insights to infer the relevance of the functional remodeling to the spectrum of neuropsychological or psychiatric profiles that cannot be fully interpreted by the tumor itself (De Baene et al., 2019; Yuan et al., 2019). Moreover, brain asymmetry and tumor laterality may account for the divergent functional disturbance and neuroplasticity induced by gliomas in a different hemisphere (Gotts et al., 2013; Zhang et al., 2016). In this study, we aimed to investigate the hemispheric specifications of glioma-induced functional remodeling and its relevance to tumor aggressiveness based on the functional connectivity and topological features of the resting-state functional networks, employing the structurally intact contralesional hemisphere as the alternative to the whole-brain maneuver.

## Materials and Methods

### Subjects

This retrospective study was approved by the local institutional review board. All participants provided written informed consent prior to the MRI examination. Preoperative resting-state fMRI (rs-fMRI) data of the subjects with cerebral glioma from March 2012 to February 2017 were consecutively retrieved, resulting in 126 subjects (male/female 72/54, aged 42.21 ± 12.74 years) for further analysis with the following criteria: 1) right-handed; 2) rs-fMRI was performed within 7 days prior to the surgery; 3) No mass effect to the contralesional hemisphere according to the neuroradiology report; 4) No previous history of neurovascular disease or psychiatric illness in the medical record; 5) No history of drug, coffee or alcohol abuse. Out of the 126 tumors, 80 were located in the left hemisphere (LH), 46 in the right hemisphere (RH). Tumors were grouped as high grade (WHO III and IV, HGG) and low grade gliomas (WHO II, LGG).

### Image Acquisition and Data Preprocessing

Rs-fMRI was performed using gradient echo-echo planar imaging (GE-EPI) sequence (Siemens Verio 3.0T, Germany) with a 12-channel phased array head coil. The major imaging parameters were TR/TE 2000/35ms, FA 90°, FOV 210 mm × 210 mm, matrix 64 × 64, slick thickness 4.0mm, 240 volumes. High resolution T1-weighted images were obtained using 3D magnetization prepared rapid gradient echo sequence (MPRAGE) with parameters of TR/TE/TI 1900/2.93/900ms, FA 9°, FOV 218 × 250 mm, in-plane resolution 0.5 × 0.5 × 1.0 mm. In addition, to identify the glioma lesions, T2 FLAIR images were obtained with parameters of TR/TE/TI 9000/96/2500ms, flip angle 150°, FOV 187 × 240 mm, in-plane resolution 0.5 × 0.5 × 2.0 mm.

Tumor region was manually delineated slice by slice on T2 FLAIR images for each subject by a neuroradiologist with 25-years of experience to generate a binary tumor mask using ITK-SNAP^1^. The tumor masks were smoothed and co-registered to the T1 weighted images. Rs-fMRI data was preprocessed using DPARSF (Data Processing Assistant for Resting-State fMRI^2^) and software package of SPM8^3^. The major steps included: 1) the first 10 volumes data were discarded for scanner calibration; 2) slice timing to remove the temporal differences between slices; 3) realignment to correct head movement; 4) T1 images and tumor mask were co-registered to the mean functional images and segmented into gray matter, white matter, and cerebrospinal fluid maps. The value of voxels within the tumor region was set to 0 while the value of voxels outside the tumor was set to 1, to generate the cost function masking image; 5) T1 images and functional images were normalized to the standard Montreal Neurological Institute template using source weighted image and resampled to 3 × 3 × 3 mm (Andersen et al., 2010); 6) spatially smoothed with a 4 mm full-width half-maximum Gaussian filter; 7) regression of nuisance variables, including Friston 24-parameter model and signals of white matter and cerebrospinal fluid (Friston et al., 1996); 8) linear trend removing; 9) band-pass filtering (0.01–0.08 Hz).

### Functional Connectivity and Topological Features

The brain of each subject was segmented into 210 cortical and 36 subcortical subregions based on the human brainnetome atlas (BN, with 123 subregions in each hemisphere) (Fan et al., 2016). These subregions of the contralesional hemisphere were defined as the nodes of functional network. Time series of voxels in each area were averaged and assigned as the node signals. Pearson correlation coefficient between each time series were calculated and defined as the edges of the network. Fisher’s *Z* transform was applied to normalize the correlation coefficient to follow standard normal distribution. This process generated a 123 × 123 functional connectivity (FC) matrix and 7,503 (123 × 122/2) edges that were assigned as FC features for each subject.

Functional connectivity matrices were converted into binary undirected graphs with an empirically defined range of sparsity (0.01 ≤ sparsity ≤ 0.34, interval = 0.01) (Zhu et al., 2017). Area under the curve (AUC) of topological feature (TF) with defined threshold range was employed to avoid the bias of any predetermined threshold (He et al., 2009; Zhu et al., 2017). Typical global and nodal TFs of functional networks were calculated using GRETNA (Graph Theoretical Network Analysis toolbox^4^): (1) Gamma (γ), Lambda (λ), Sigma (σ), the clustering coefficient (Cp) and the shortest length (Lp) that quantitate the “small-worldness” of network; (2) the global efficiency (Eg) and the local efficiency (Eloc) that characterize the ability of the network to integrate and separate information; (3) the Betweeness centrality (Bc), Degree centrality (Dc), nodal global efficiency (NEg) and nodal local efficiency (NEloc) that characterize the importance of each node in the network to process information (Watts and Strogatz, 1998; Humphries and Gurney, 2008; Zhu et al., 2017). This process generated seven global TFs (aGamma, aLambda, aSigma, aCp, aLp, aEg, and aEloc) and 492 local TFs (4 × 123, aBc, aDc, aNEg, and aNEloc of 123 nodes) for each subject.

### Feature Selection and Multivariate Logistic Regression Analysis

**FIGURE 1:**
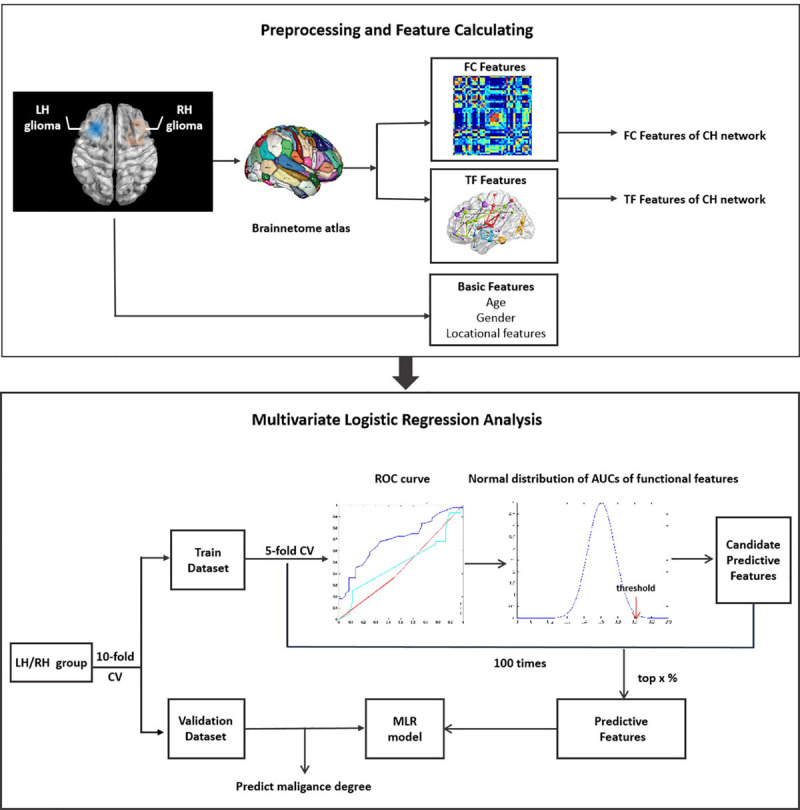
Illustration of the workflow of data analysis. LH, tumor located in left hemisphere; RH, tumor located in right hemisphere; CH, contralesional hemisphere; LGG, low grade glioma; HGG, high grade glioma; CV, cross validation.

Data was divided into training and validation datasets. The process of feature selection includes three steps (Figure 1). (1) Firstly, the training dataset was further subdivided into two subgroups of train-train dataset and train-test dataset with the inner five-fold cross validation (CV). The ROC curve and AUC analysis were utilized to estimate the classification performance of each single functional or demographic feature in discriminating LGGs and HGGs. (2) Secondly, the normal distribution function of all FC features or TFs was estimated. One-sided confidence interval (CI) was set to threshold the AUCs. Features with high AUC values in discriminating LGGs and HGGs in the train-train dataset were selected as candidate predictive features. (3) Finally, the inner CV process was repeated 100 times and the frequency of each feature being selected as a candidate feature was recorded and ranked. The candidate predictive features with top frequencies of being selected were identified as predictive features, the corresponding brain regions were singled out as the most sensitive regions to tumor-induced functional remodeling.

Given the high dimension of functional features, a series of one-sided CIs of AUC values (from 95 to 99.9%) were employed to identify candidate predictive features. A range of thresholds were set from the top 1 to top 20% for the selection of predictive features from the candidate features. The process of feature selection was illustrated in Supplementary Figure 1.

A multivariate logistic regression (MLR) model was constructed on the training dataset based on different subsets of predictive features selected with different thresholds. The classification performance of the MLR model was evaluated on training and validation datasets separately by ROC analysis with 10-fold CV.

The aforementioned process was applied to the LH and RH groups separately. Synthetic minority oversampling technique (SMOTE) was employed to tackle the data imbalance (Chawla et al., 2002).

### Hemispheric Specificity of Functional Remodeling

In order to investigate the potential hemisphere-specific effect, MLR models constructed based on the training dataset of the LH group were validated by both the validation dataset of the LH group and the RH group, and vice versa for the MLR models constructed with the training dataset of the RH group. The classification performance of the MLR model on two kinds of validation datasets were quantified by ROC and AUC analysis and the intergroup difference was assessed by two-tailed *t*-test analysis.

## Results

**FIGURE 2:**
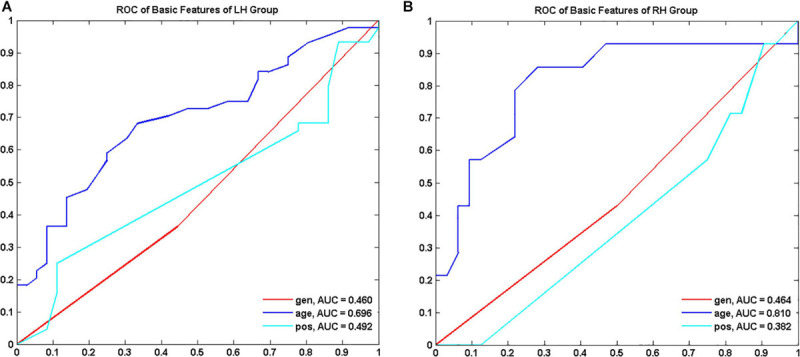
Receiver operating characteristic curve (ROC) of demographic features to discriminate low grade and high-grade gliomas for LH **(A)** and RH group **(B)**, respectively. LH, tumor located in left hemisphere; RH, tumor located in right hemisphere; AUC, area under the curve.

Demographics and tumor information are summarized in Table 1. The LH group with tumors in the LH included 36 LGGs (male/female 20/16, aged 38.54 ± 10.88 years) and 44 HGGs (male/female 28/16, aged 45.06 ± 13.21 years). The RH group with tumors in the **RH** included 32 LGGs (male/female 16/16, aged 39.48 ± 10.46 years) and 14 HGGs (male/female 8/6, aged 51.25 ± 17.81 years). Sex distribution was comparable between LGGs and HGGs (Chi-square test, *p* > 0.05). Age of subjects with HGG was significantly larger than that of subjects with LGG for both LH and RH groups (Mann–Whitney *U* test, *p* < 0.05). The ROC curves of age, sex, and tumor location to differentiate LGGs and HGGs were shown in Figure 2. Age was identified as one of the predictive features outperforming sex and tumor location in malignant grade discrimination (LH: AUC = 0.696, RH: AUC = 0.810).

**TABLE 1:** Summary of the demographic information

**WHO Grade**		**Cellular Type**	**Sex (male/female)^*a*^**		**Age(mean ± SD)^*b*^**	
			**LH**	**RH**	LH*	RH*
**LGG**	**II**	Astrocytoma (*n* = 30)	20/16	16/16	38.54 ± 10.88	39.48 ± 10.46
		Oligodendroglioma (*n* = 16)				
		Oligoastrocytomas (*n* = 22)				
**HGG**	**III**	Anaplastic astrocytoma (*n* = 19)	28/16	8/6	45.06 ± 13.21	51.25 ± 17.81
		Anaplastic oligodendroglioma (*n* = 1)				
		Anaplastic oligoastrocytomas (*n* = 8)				
	**IV**	Glioblastoma (*n* = 30)				

*^*a*^Chi-square test.^*b*^Mann–Whitney *U* test.^∗^Significant difference between LGGs and HGGs with *p* < 0.05.LGG, low grade glioma; HGG, high grade glioma; LH, tumor located in left hemisphere; RH, tumor located in right hemisphere.*

Both predictive FC features and TFs varied with tumor hemisphere. The performance of malignant grade classification of the MLR models was summarized in Supplementary Material in detail (Supplementary Figures 2–5). MLR models with age feature significantly outperformed the MLR models without age in differentiating LGGs from HGGs for both LH and RH groups (paired *t*-test, *p* < 0.05) (Supplementary Table 3).

### Predictive FC Features

**FIGURE 3:**
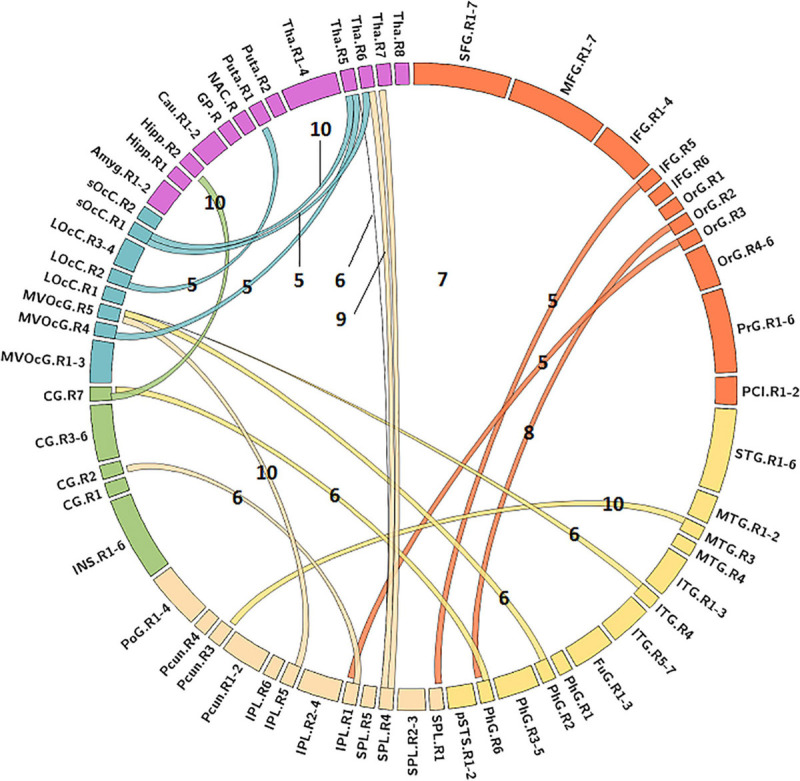
Predictive functional connectivity features of LH group. LH, tumor located in left hemisphere; the number label of ribbons represented the frequency of the functional connectivity feature being selected as predictive feature during 10-fold cross validation process; the full names of subregions are summarized in the Supplementary Table 4.

The MLR model constructed with predictive FC features of LH group achieved the best performance of malignant grade estimation with AUC of 0.853 ± 0.079 on validation dataset (one-sided CI = 99.6%, top 5%) (Table 2). Subsets of predictive FC features changed during the 10-fold CV process with 50 spatially distributed FC features defined as predictive (Supplementary Table 1), among which 17 FC features were selected as predictive feature for at least five times (Figure 3). These predictive FC features mainly involve the functional connectivity between thalamus (Tha.R8_5/6)-occipital lobe, thalamus (Tha.R8_5/6/7)-postcentral area of superior parietal lobe (SPL.R5_4), and between distributed areas of cortices.

**TABLE 2:** Performance of multivariate logistic models to predict malignancy degree of gliomas.

	**Features**	**One-sided CI (%)**	**Top (%)**	**AUC (mean ± std)**			Two-sample *t*-test (*p* value, FDR)*
				**Train dataset**	**Validation dataset**	**Dataset of another group**	
LH	Age + FC	99.6	5	0.996 ± 0.003	0.853 ± 0.079	0.626 ± 0.082	<0.05
	Age + TF	98	15	0.896 ± 0.016	0.788 ± 0.150	0.629 ± 0.040	<0.05
RH	Age + FC	99.7	5	1.000 ± 0.000	1.000 ± 0.000	0.577 ± 0.028	<0.05
	Age + TF	99	20	0.968 ± 0.010	0.897 ± 0.165	0.640 ± 0.032	<0.05

*CI: confidence interval to select candidate predictive features. Top: features with top high frequencies of being selected as candidate predictive features. ^∗^Significant intergroup difference between MLR model performance on validation dataset and the performance on dataset of another group.*

**FIGURE 4:**
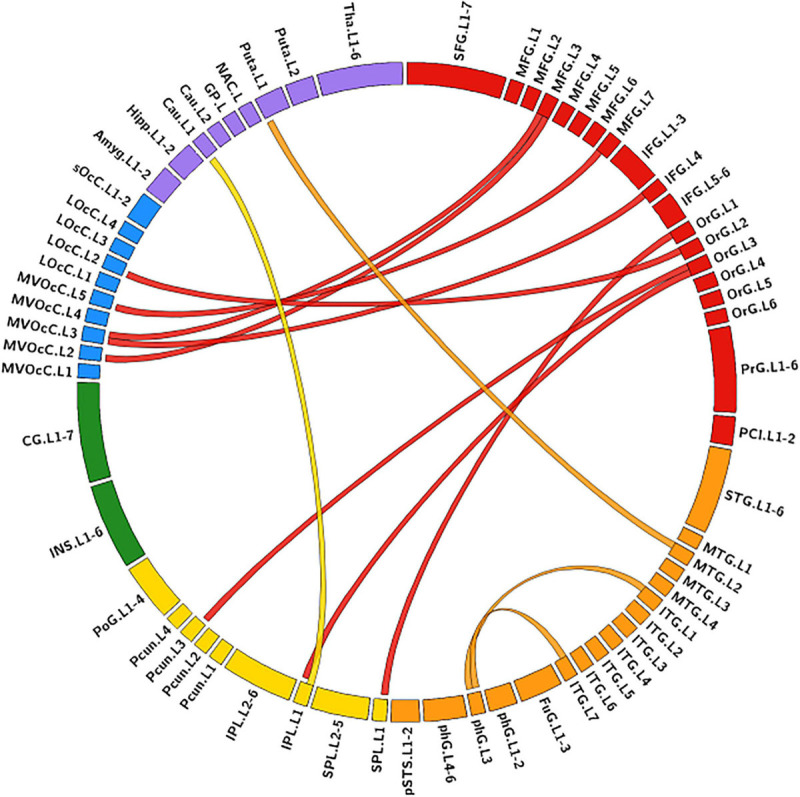
Predictive functional connectivity features of RH group. RH, tumor located in right hemisphere; the full names of subregions are summarized in the Supplementary Table 4.

The MLR model constructed with predictive FC features of RH group achieved an AUC of 1.000 ± 0.000 on validation dataset (one-sided CI = 99.7%, top 5%) (Table 2). Twelve FC features were defined as predictive features (Figure 4), mainly involving FC between medioventral occipital cortex (MVOcC.L)-middle frontal gyrus (MFG.L), FC between inferior temporal gyrus (ITG.L7_1/7)-parahippocampal (PhG.L6_3), FC between orbital gyrus (OrG.L)-precuneus (PCun.L), superior and inferior parietal lobe (SPL.L and IPL.L), FC between rostral area of middle temporal gyrus (MTG.L4_2)-ventromedial putamen (Puta.L2_1), and FC between caudal area of inferior parietal lobe (IPL.L6_1)-ventral caudate (Cau.L2_1). The subsets of predictive FC features of RH group were summarized in Supplementary Table 2.

### Predictive Topological Features

**FIGURE 5:**
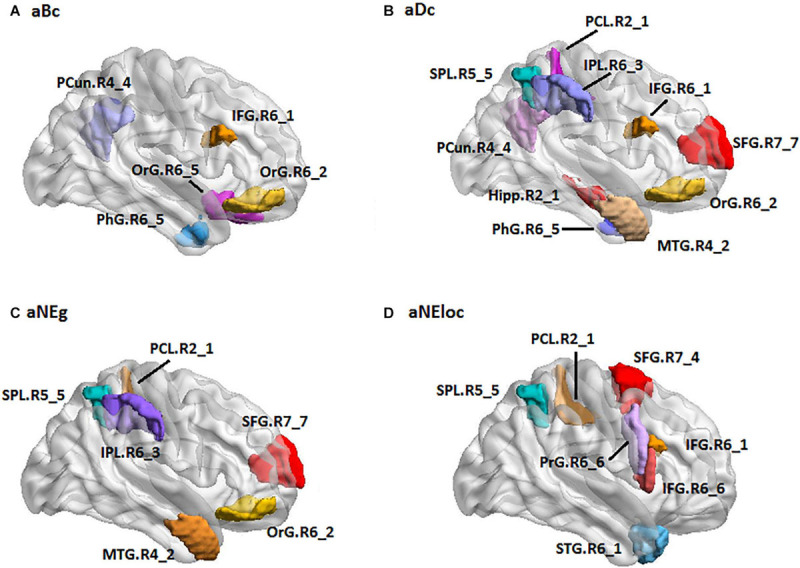
The spatial distribution of brain regions with nodal topological features selected as predictive features for LH group [**(A)**: aBc, **(B)**: aDc, **(C)**: aNEg, **(D)**: aEloc]. LH: tumor located in left hemisphere; the full names of subregions are summarized in the Supplementary Table 4.

For the LH group, the MLR model constructed with predictive TFs achieved the best performance of malignancy estimation with an AUC of 0.788 ± 0.150 on the validation dataset (one-sided CI = 98%, top 15%) (Table 2). Twenty-eight nodal TFs were selected as predictive features and the corresponding cerebral regions mainly involved orbital gyrus (OrG.R6_2/5), superior frontal gyrus (SFG.R7_4/7), inferior frontal gyrus (IFG.R6_1/6), paracentral lobule (PCL.R2_1), intraparietal area of superior parietal lobule (SPL.R5_5), rostrodorsal area of inferior parietal lobule (IPL.R6_3), rostral area of hippocampus (Hipp.R2_1), medial area of superior temporal gyrus (STG.R6_1), PCun.R4_4, and PhG.R6_5 (Figure 5).

**FIGURE 6:**
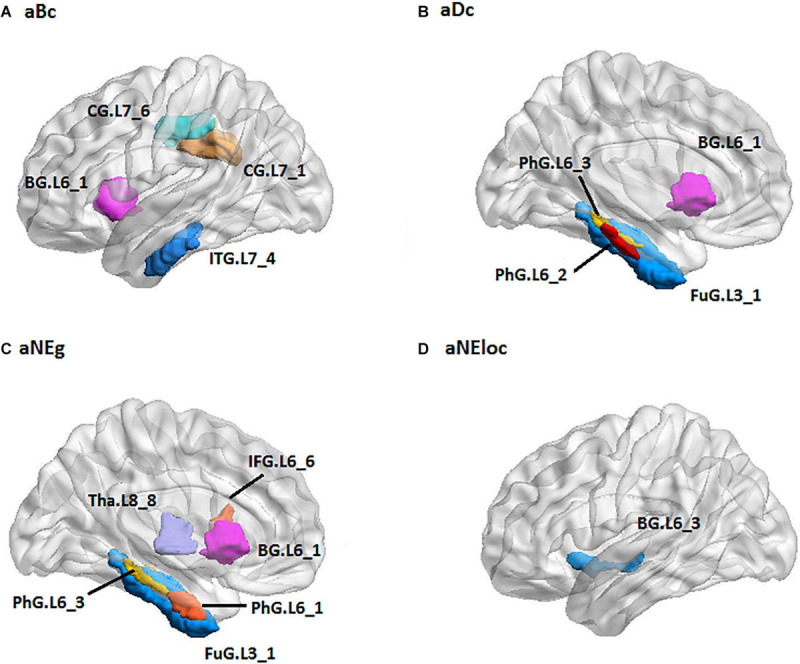
The spatial distribution of brain regions with nodal topological features selected as predictive features for RH group [**(A)**: aBc, **(B)**: aDc, **(C)**: aNEg, **(D)**: aEloc]. RH: tumor located in right hemisphere; the full names of subregions are summarized in the Supplementary Table 4.

For the RH group, the MLR model constructed with predictive TFs achieved the best performance of malignancy estimation with an AUC of 0.897 ± 0.165 on the validation dataset (one-sided CI = 99%, top 20%) (Table 2). Sixteen nodal TFs were selected as predictive features and the corresponding cerebral regions were centralized located in the inferior and medial part of left temporal lobe (rostroventral area of left fusiform gyrus: FUG.L3_1; PhG.L6_1/2/3; intermediate lateral area of inferior temporal gyrus: ITG.L7-4), dorsal and caudal area of cingulate gyrus (CG.L7_1/6), basal ganglia (ventral caudate BG.L6_1 and nucleus accumbens BG.L6_3), ventral area of inferior frontal gyrus (IFG.L6_6), and lateral pre-frontal thalamus (Tha.L8_8) (Figure 6).

### Hemisphere-Specific Effect of Gliomas on Functional Remodeling

Predictive FC features and TFs of the functional network of contralesional hemisphere showed distinct hemisphere-specific distribution (Figures 3–6). MLR models constructed based on the training dataset and predictive features of LH group were validated by the validation dataset of the LH group and the RH group. The AUC values of the validation dataset of the LH group were significantly higher than those of the dataset of the RH group (two-tailed *t*-test, *p* < 0.05, FDR) (Table 2). The MLR models constructed based on the training dataset of the RH group were also validated by the validation dataset of the RH group and the dataset of the LH group, respectively. MLR models showed significantly better classification performance on the validation dataset of the RH group, as compared with the dataset of the LH group (two-tailed *t*-test, *p* < 0.05, FDR) (Table 2).

## Discussion

Growth of glioma may trigger neural plasticity at variable levels, which underpins the functional remodeling and clinical profiles (Derks et al., 2017; Fox and King, 2018). This study investigated the functional remodeling and its relevance to the malignancy grade of glioma based on the contralesional functional network. Functional remodeling patterns were found to be hemisphere-specific and highly predictive of the biological aggressiveness of glioma.

### Physiological Underpinnings of Functional Remodeling Induced by Glioma

Neuroplasticity is hypothesized to pivot functional remodeling, but the exact mechanisms were not completely known (Duffau, 2014). Recent study found that the interaction of glioma cells and active neurons changes the excitability of the brain, thus may alter the neurovascular coupling and subsequently results in varied functional connectivity (Venkatesh et al., 2019). In addition, the growth of glioma changes the topological features of cerebral vessel network, possibly by hemodynamic remodeling, extended effect of aberrant metabolite and neurotransmitter, or other unknown mechanisms (Bowden et al., 2018; Hahn et al., 2019). Functional plasticity occurs at various levels in response to glioma growth with different kinetics (Duffau, 2014). The gradual growth of LGGs leaves time for the brain to reconstruct the network integrity, partially by recruiting the contralesional areas more efficiently than the fast growing HGGs (Duffau, 2014). Tantillo et al. found an increase in slow network oscillations in mouse visual cortex with GL261 glioma implantation (Tantillo et al., 2019). These findings suggest a cellular and molecular basis underpinning the network plasticity during the dynamic of glioma progress. As a result of glioma-neuron interaction, the pattern of functional remodeling may represent a part of the neoplastic entity rather than the secondary consequence of cerebral gliomas.

### Predictive Performance of Functional Features

Human brain network features small-worldness and high efficiency of parallel information processing with global integration and local specialization (Achard et al., 2006). FC features and TFs characterize functional networks from different aspects and offer complementary information of functional remodeling. FC features focus on the direct measurement of the synchronicity between cerebral regions, while TFs quantify the nodal importance and the efficiency of information processing over the network at both global and local levels (Watts and Strogatz, 1998). Abundant studies indicate that brain pathology is associated with FC alterations, nodal TF disturbance and efficiency decline of parallel information processing (Mallela et al., 2016; Derks et al., 2017; Zhu et al., 2017). In this study, both predictive FC features and TFs showed efficient ability signifying the malignancy degree of gliomas. The MLR model with predictive FC features resulted in a better malignancy grade differentiation as compared with MLR-TFs. As feature selection algorithms and classification models are designed with different criteria, their performances vary in pattern recognition (Lee, 2009). This may suggest a better sensitivity of linear combination of connectivity features in this study in capturing the biological aggressiveness of glioma. Future work with advanced classification models and improved feature selection algorithms combining filter and wrapper methods may further improve the performance of TFs in glioma characterization.

### Hemispheric Specification of Functional Remodeling

Both predictive features and MLR models showed hemispheric specification between LH and RH group in this study. For LH group, the cerebral regions with predictive FC features were sparsely distributed and changed with the trianing dataset, and the regions with predictive TFs were located in the frontal and parietal lobes which are mainly associated with higher cognitive and emotional functions and involved in the default mode network and frontal-parietal network (Yeo et al., 2011; Kocher et al., 2020). Altered topological features of these regions were found to be associated with severe cognitive deficits of HGGs as compared with LGGs (Noll et al., 2014), especially for subjects with tumors located in the LH (Hendrix et al., 2017). In contrast, the predictive FC features of RH group stably involved the FC between MTG.L-MVOcC.L and FC between OrG.L-parital lobule (PCun.L/IPL.L/SPL.L), unvarying with the training dataset. The predictive TFs of the RH group were centralized in the parahippocampus and basal ganglia. These areas are mainly associated with memory processing, visuospatial attention, and face recognition (Rolls, 2004; Baumann and Mattingley, 2016; Weiner and Zilles, 2016). As the healthy human brain is functionally lateralized (Gotts et al., 2013), the altered network features with hemispheric specifications found in this study may partially underpin 1) the scope of neuro-psychological symptoms that cannot be solely explained by the tumor itself, and 2) the lower self-perceived quality of life of glioma subjects (Moody et al., 2005), although the mechanisms underpinning the hemispheric specification of the functional remodeling remain unclear. Factors including the lobar and hemispheric heterogeneity of glioma incidence and the dynamic lateralization of cerebral blood flow may partially substrate the different patterns of glioma-induced functional remodeling (Moody et al., 2005; Larjavaara et al., 2007). As functional connectivity and network topology disturbances precede the onset of some neurological diseases (Harrington et al., 2015), therapeutic schemes targeting the hemisphere-specific functional remodeling may provide a new opportunity to innovate the patient management and further optimize the disease outcome of glioma.

### Limitations

There are limitations for our study. First, it is elusive to avoid overfitting with the limited sample size. The extraordinary high AUC of MLR model in predicting the malignancy grade of gliomas of the RH group may be attributable to the introduction of simulation samples by SMOTE. A hemisphere-specific analysis with a larger cohort and advanced machine learning model may yield a more reliable classification performance. Second, functional networks of the human brain are believed to be scale-free with distinct importance for each subregion. Functional impairment may vary with different nodes being attacked. Network statistical analysis based on more detailed anatomical specifications would be helpful in clarifying the disturbance induced by glioma with better functional representations. Lastly, molecular pathologies were not available for all the subjects, as the histopathology analysis was largely conducted before WHO issued the new guidelines on the classification of central nervous system tumor (Louis et al., 2016). Future work integrating the morphological, functional and molecular data would enable a more intensive comprehension of the glioma-induced functional remodeling with malignancy relevance with histopathological and genetic specifications.

## Conclusion

Focal glioma induces extensive functional disturbance with hemispheric specification. The clinical and biological effects of focal glioma may need to be interpreted in terms of the global changes. Disturbance in the functional network of rs-fMRI may substrate the pathophysiological mechanism of tumor progress at the tissue level. A network approach with machine learning provides a novel pathway and potential biomarker for high-dimensional imaging data analysis toward better tumor characterization of glioma.

## Data Availability Statement

The raw data supporting the conclusions of this article will be made available by the authors, without undue reservation.

## Ethics Statement

The studies involving human participants were reviewed and approved by Shenzhen Institutes of Advanced Technology Institutional Review Board. The patients/participants provided their written informed consent to participate in this study.

## Author Contributions

SC and LZ contributed to data processing, manuscript drafting, conceived and designed the research project, approved the study, and guaranteed the scientific integrity of the manuscript. ZS and LC contributed to the glioma surgery and clinical data analysis. CJ, KW, and LA contributed to the MR imaging data quality insurance. All authors contributed to the article and approved the submitted version.

## Conflict of Interest

The authors declare that the research was conducted in the absence of any commercial or financial relationships that could be construed as a potential conflict of interest.

## Footnotes

1 http://www.itksnap.org

2 http://www.restfmri.net

3 http://www.fil.ion.ucl.ac.uk/spm

4 https://www.nitrc.org/projects/gretna
