# Supplementary material for: Hemisphere-Specific Functional Remodeling and Its Relevance to Tumor Malignancy of Cerebral Glioma Based on Resting-State Functional Network Analysis
Source: Front Neurosci. 2021 Jan 13;14:611075. doi: 10.3389/fnins.2020.611075 (PMC7838505; doi:10.3389/fnins.2020.611075)
Supplement: Supplementary file 1 [file Table_1.pdf]

Supplementary Material

Hemisphere-Specific Functional Remodeling and Its Relevance to Tumor Malignancy of Cerebral Glioma Based on Resting-State Functional Network Analysis

Siqi Cai[1,2], Zhifeng Shi[3], Chunxiang Jiang[1,2], Kai Wang[4], Liang Chen[3], Lin Ai[4] and Lijuan Zhang[1]*

1 Paul. C. Lauterbur Research Centers for Biomedical Imaging, Shenzhen Institutes of Advanced Technology, Chinese Academy of Sciences, Shenzhen, China,

2 University of Chinese Academy of Sciences, Beijing, China,

3 Department of Neurosurgery, Huashan Hospital of Fudan University, Shanghai, China,

4 Beijing Neurosurgical Institute, Beijing Tiantan Hospital, Capital Medical University, Beijing, China

1 Supplementary Figures

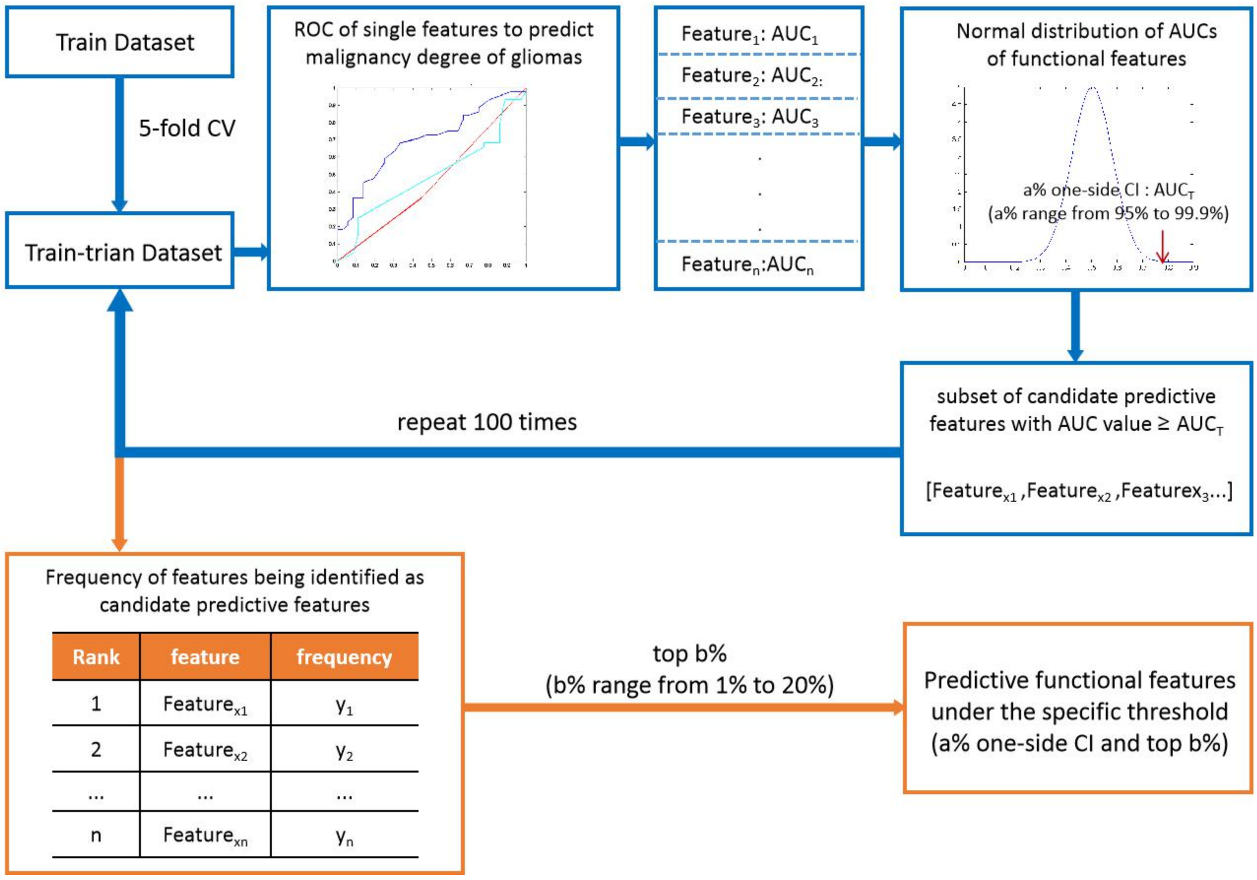

Supplementary Figure 1. Illustration of the workflow of feature selection.

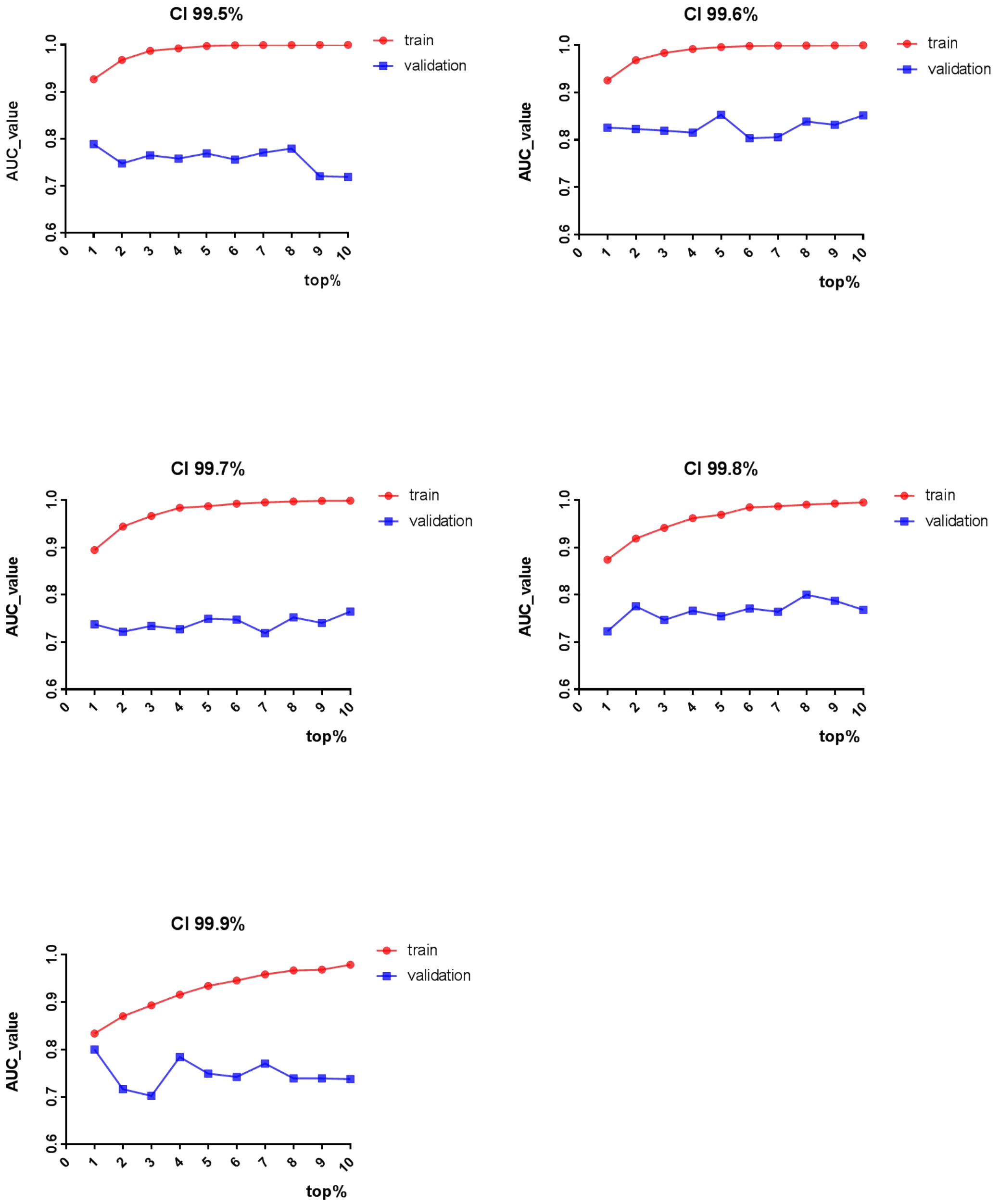

Supplementary Figure 2. The classification performance of multivariate logistic regression (MLR) model on different subsets of predictive functional connectivity features of LH group. A series of threshold a series of one-side CIs of AUC values (from 99.5% to 99.9%) were applied to identify candidate predictive features, from which the candidate features with top 1% to 10% chances being selected were finally determined as predictive features. By 99.6% one-side CI for defining candidate predictive features and from which the features with top 5% chances of being selected in 100 times inner 5-fold CV process were determined as predictive features, the MLR model of LH group achieved the best performance of malignant grade estimation with AUC of 0.853±0.079 on validation dataset.

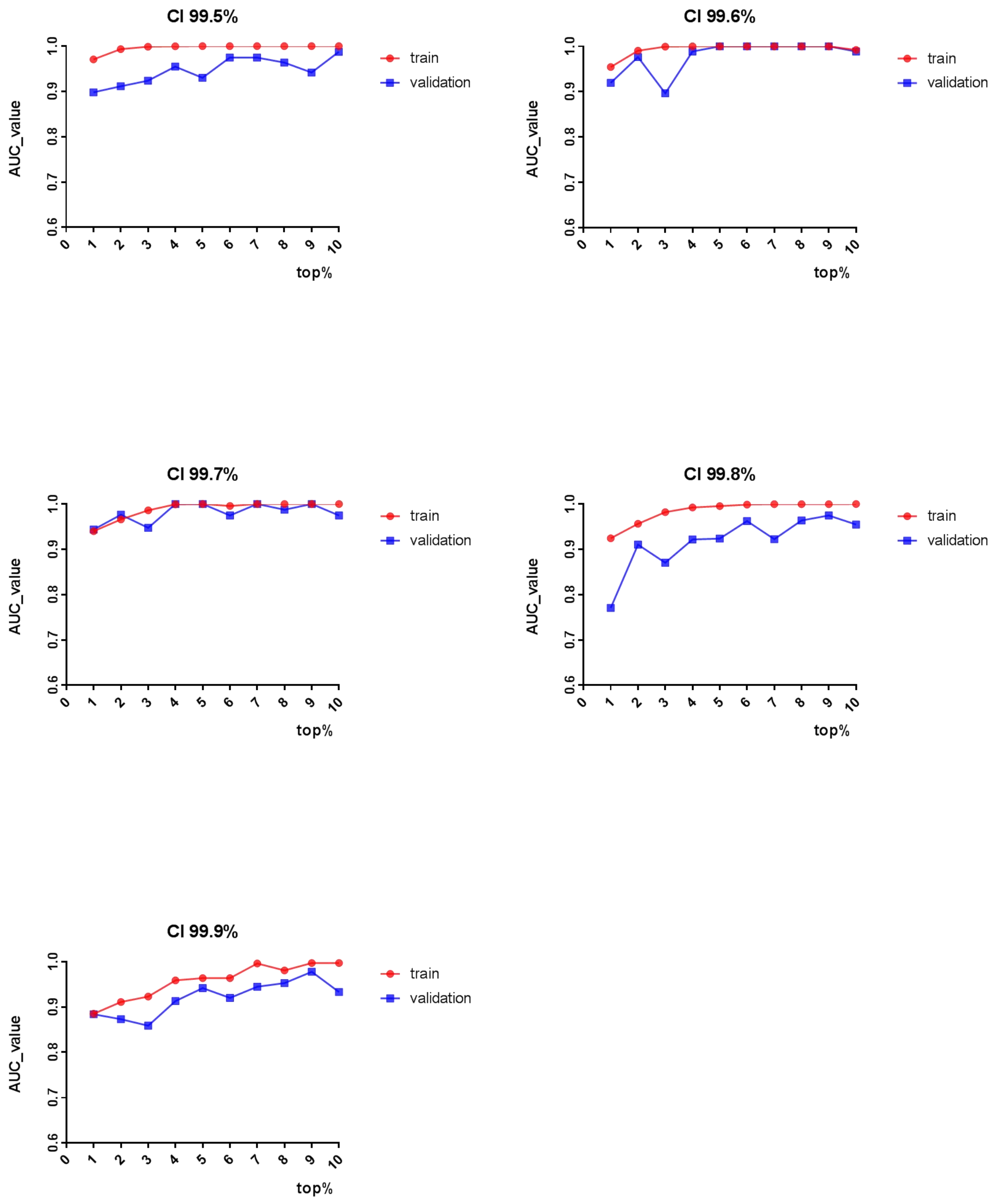

Supplementary Figure 3. The classification performance of MLR model on different subsets of predictive functional connectivity features of RH group. A series of threshold a series of one-side CIs of AUC values (from 99.5% to 99.9%) were applied to identify candidate predictive features, from which the candidate features with top 1% to 10% chances being selected were finally determined as predictive features. By 99.7% one-side CI for defining candidate predictive features and from which the features with top 5% chances of being selected in 100 times inner 5-fold CV process were determined as predictive features, the MLR model of RH group achieved the best performance of malignancy estimation with AUC of 1.00±0.00 on validation dataset.

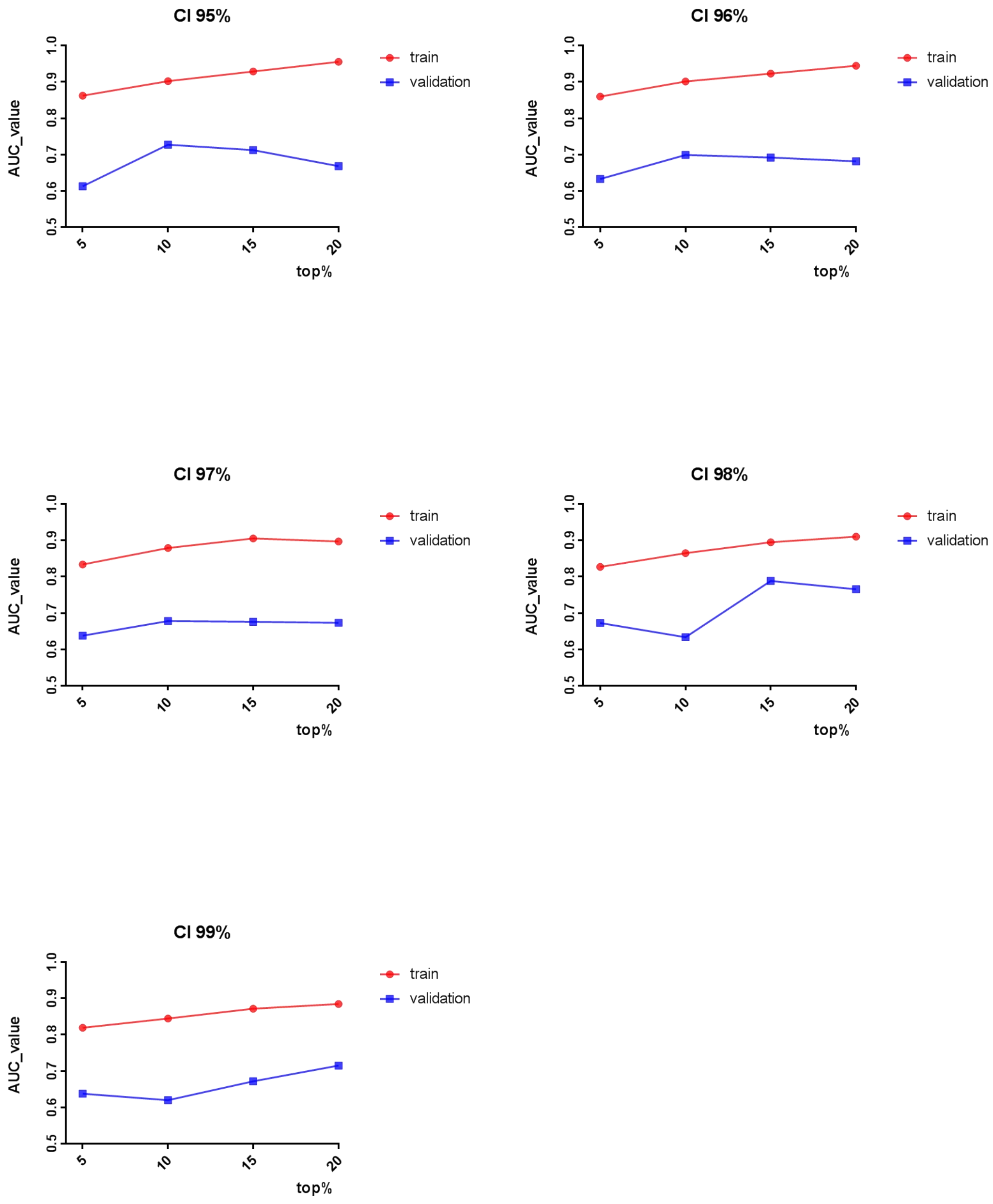

Supplementary Figure 4. The classification performance of multivariate logistic regression model on different subsets of predictive topological features of LH group. A series of threshold a series of one-side CIs of AUC values (from 95% to 99%) were applied to identify candidate predictive features, from which the candidate features with top 5% to 20% chances being selected were finally determined as predictive features. By 98% one-side CI AUCs for defining candidate predictive features and from which features with top 15% chances of being selected in 100 times inner 5-fold CV were determined as predictive features, the MLR model achieved the best performance of malignancy estimation with AUC of 0.761±0.126 on validation dataset.

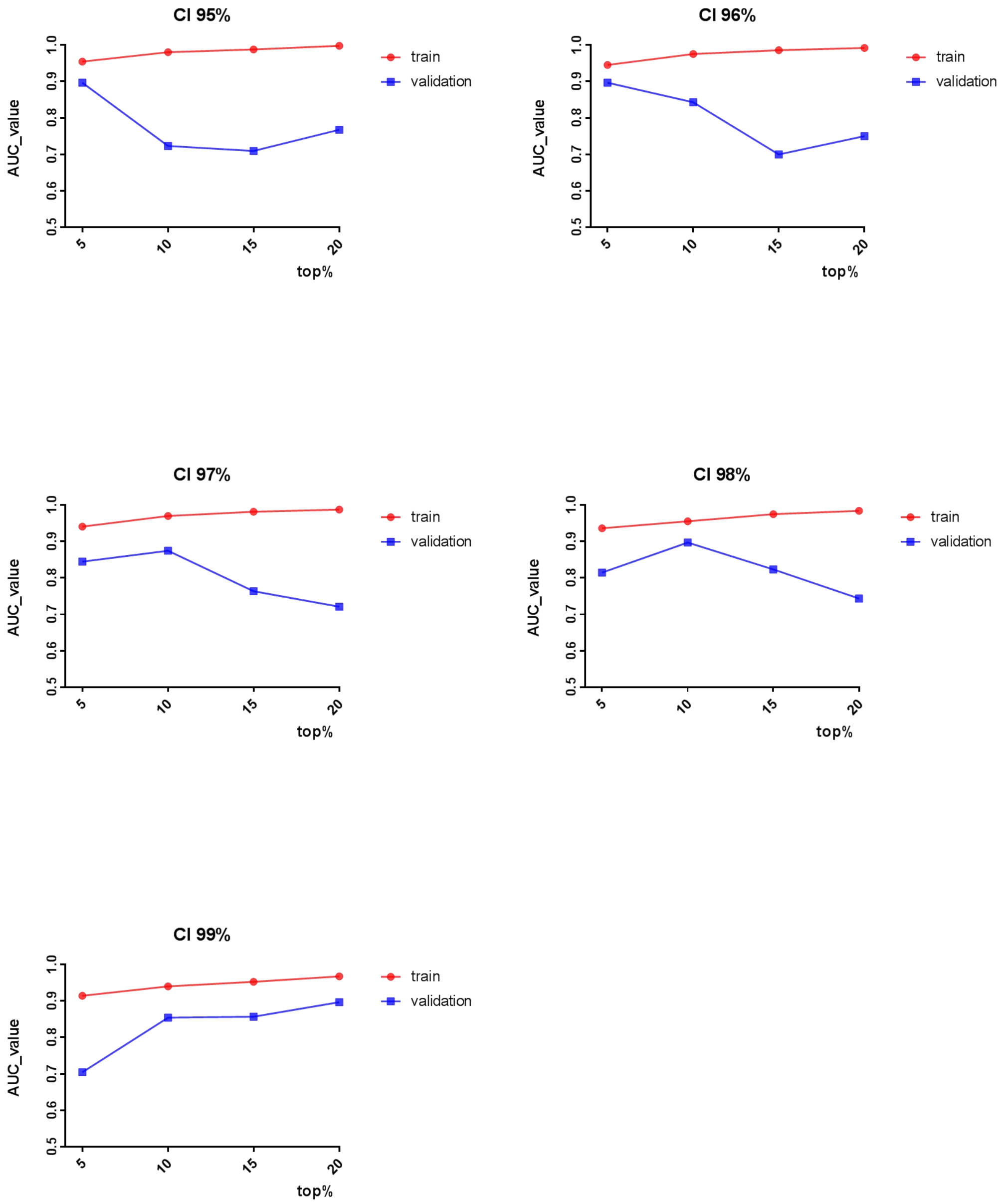

Supplementary Figure 5. The classification performance of multivariate logistic regression model on different subsets of predictive topological features of RH group. A series of threshold a series of one-side CIs of AUC values (from 95% to 99%) were applied to identify candidate predictive features, from which the candidate features with top 5% to 20% chances being selected were finally determined as predictive features. By 99% one-side CI of AUCs for defining candidate predictive features and from which features with top 20% chances of being selected in 100 times inner 5-fold CV were identified as predictive features, the MLR model achieved the best performance of malignancy estimation with AUC of 0.897±0.165 on validation dataset.

2 Supplementary Tables

Table 1. Subsets of Predictive Functional Features for LH Group

	CI (%)	Top (%)	Subset	**Subsets of Predictive Features**
**FC**	**99.6**	**5**	1	OrG.R6_3-IPL.R6_1; PhG.R6_2-STG.R6_3/6_4/MVOcC.R5_5; sOccG.R2_1-Tha.8_5/8_6; MVOcC.R5_5-ITG.R7_4/IPL.R6_5; MVOcC.R5_4-Tha.R8_5/6; MTG.R4_3-PCun.R4_3; FuG.R3_2-Tha.R8_1/8_6/8_7; CG.R7_7-PhG.R6_6/Hipp.R2_2; SPL.R5_4-Tha.R8_5/8_6/8_7
			2	OrG.R6_2-PhG.R6_6; OrG.R6_3-IPL.R6_1; FuG.R3_3-Tha.R8_7; MTG.R4_3-PCun.R4_3; MVOcC.R5_5-MTG.R4_3/ITG.R7_4/PhG.R6_2/IPL.R6_5; IPL.R6_1-PCun.R4_3/CG.R7_2; IPL.R6_3-PCun.R4_2; SPL.R5_4-IPL.R6_4/BG.R6_1/Tha.R8_6/8_7; CG.R7_7-Hipp.R2_1/2_2; LOcC.R4_2-BG.R6_5; sOccG.R2_1-Tha.R8_5
			3	IFG.R6_5-ITG.R7_2/7_5/SPL.R5_1;PhG.R6_6-OrG.R6_2/CG.R7_7; MTG.R4_3-PCun.R4_3; IPL.R6_1-OrG.R6_3/CG.R7_2; INS.R6_3-ITG.R7_5/SPL.R5_1; FuG.R3_2-Tha.R8_7; CG.R7_7-MTG.R4_2/PhG.R6_6/Hipp.R2_2; SPL.R5_4-Tha.R8_6/8_7; sOccG.R2_1-Tha.R8_5; MVOcC.R5_5-ITG.R7_4/IPL.R6_5
			4	IFG.R6_5-SPL.R5_1; OrG.R6_2-PhG.R6_6; OrG.R6_6-PhG.R6_6/PCun.R4_4; MTG.R4_3-PCun.R4_3; CG.R7_7-PhG.R6_3/6_6/Hipp.R2_2; SPL.R5_4-Tha.R8_5/8_6/8_7; IPL.R6_5-MVOcC.R5_5; LOcC.R4_2-BG.R6_5; Tha.R6_5-SFG.R7_5/sOccG.R2_1
			5	IFG.R6_5-ITG.R7_5/SPL.R5_1; IFG.R6_6-ITG.R7_5; OrG.R6_2-PhG.R6_6; OrG.R6_3-PhG.R6_5; MTG.R4_3-PCun.R4_3; SPL.R5_1-INS.R6_6;CG.R7_7-IPL.R6_1/Hipp.R2_2; SPL.R5_4-IPL.R6_4/Tha.R8_5/8_6/8_7; MVOcC.R5_5-ITG.R7_4/PhG.R6_2/IPL.R6_5; LOcC.R4_2-BG.R6_5; sOCCG.R2_1-Tha.R8_5/8_6
			6	MTG.R7_1-LocC.R4_3; OrG.R6_6-PCun.R4_4;STG.R6_4-PhG.R6_2; ITG.R7_5-INS.R6_3; PhG.R6_6-OrG.R6_2/CG.R7_7; MTG.R4_3-PCun.R4_3; SPL.R5_4-Tha.R8_6; CG.R7_2-MTG.R4_2/IPL.R6_1/Hipp.R2_2; CG.R7_7-Hipp.R2_2; FuG.R3_2-Tha.R8_1; MVOcC.R5_5-MTG.R4_3/ITG.R7_4/IPL.R6_5/Hipp.R2_1; Tha.R8_5-MVOcC.R5_3/sOccG.R2_1
			**7**	OrG.R6_2-PhG.R6_6; MTG.R4_3-PCun.R4_3; CG.R7_7-PhG.R6_3/6_6/IPL.R6_1/Hipp.R2_1/2_2; FuG.R3_3-Tha.R8_7; SPL.R5_4-Tha.R8_5/8_6/8_7; IPL.R6_1-CG.R7_2; MVOcC.R5_4-Tha.R8_5; MVOcC.R5_5-PhG.R6_2/IPL.R6_5; LOcC.R4_2-BG.R6_5; sOccG.R2_1-Tha.R8_5/8_6
			**8**	OrG.R6_3-PhG.R6_5/IPL.R6_1; MTG.R4_3-PCun.R4_3; SPL.R-INS.R6_6; CG.R7_2-MTG.R4_2/IPL.R6_1; CG.R7_7-MTG.R4_2/IPL.R6_1; MVOcC.R5_5-IPL.R6_5; Tha.R8_5-SPL.R5_4/MVOcC.R5_4/sOccG.R2_1; Tha.R8_6-SPL.R5_4; Tha.R8_7-FuG.R3_2
			**9**	IFG.R6_5-SPL.R5_1; OrG.R6_2-PhG.R6_1; OrG.R6_6-PCun.R4_4; MTG.R4_2-CG.R7_2/7_7; MTG.R4_3-PCun.R4_3; SPL.R5_4-IPL.R6_4/Tha.R8_5/8_6/8_7; IPL.R6_5-MVOcC.R5_5; CG.R7_7-Hipp.R2_2; Tha.R8_1-FuG.R3_2; Tha.R8_5-MVOcC.R5_4/sOccG.R2_1; Tha.R8_6-MVOcC.R5_4/sOccG.R2_1
			**10**	OrG.R6_2-PhG.R6_6; OrG.R6_6-PCun.R4_4; PrG.R6_5-SPL.R5_1; MTG.R4_3-PCun.R4_3; SPL.R5_1-IFG.R6_5/INS.R6_6; IPL.R6_1-OrG.R6_3/CG.R7_2; LOcC.R4_2-BG.R6_5; CG.R7_7-PhG.R6_6/Hipp.R2_1/2_2; MVOcC.R5_5-MTG.R4_3/ITG.R7_4/PhG.R6_2/IPL.R6_5; sOccG.R2_1-Tha.R8_5/8_6

**TF**	**98**	**15**	**1**	aBc: OrG.R6_2, PhG.R6_5, PCun.R4_4; aDc: OrG.R6_2, PCL.R2_1, SPL.R5_5;<br>aNEg: OrG.R6_1, PCL.R2_1, SPL.R5_5; aNEloc: SFG.R7_4, IFG.R6_1, PrG.R6_6
			**2**	aBc: PhG.R6_5, PCun.R4_4; aDc: SFG.R7_7, OrG.R6_2, MTG.R4_2, IPL.R6_3;<br>aNEg: SFG.R7_7,OrG.R6_2, MTG.R4_2, IPL.R6_3; aNEloc: IFG.R6_1
			**3**	aBc: OrG.R6_5, PhG.R6_5; aDc: SFG.R7_7, OrG.R6_2, IPL.R6_3;<br>aNEg: SFG.R7_7,OrG.R6_2; aNEloc : IFG.R6_1, PrG.R6_6
			**4**	aBc: PCun.R4_4; aDc: SFG.R7_7, OrG.R6_2, MTG.R4_2, IPL.R6_3;<br>aNEg: SFG.R7_7, OrG.R6_2, IPL.R6_3; aNEloc: PrG.R6_6, SPL.R5_5
			**5**	aBc: PhG.R6_5, PCun.R4_4; aDc: OrG.R6_2, SPL.R5_5, IPL.R6_3, Hipp.R2_1;<br>aNEg: OrG.R6_2, SPL.R5_5, IPL.R6_3; aNEloc: PrG.R6_6, PCL.R2_1,STG.R6_1
			**6**	aBc: PhG.R6_5, PCun.R4_4; aDc: OrG.R6_2, IPL.R6_3, Hipp.R2_1; aNEg: OrG.R6_2, IPL.R6_3;<br>aNEloc: IFG.R6_6, PrG.R6_6, PCL.R2_1
			**7**	aBc: PhG.R6_5, IPL.R6_3, PCun.R4_4; aDc: OrG.R6_2, SPL.R5_5,IPL.R6_3, Hipp.R2_1;<br>aNEg: OrG.R6_2, IPL.R6_3; aNEloc: IFG.R6_1, PrG.R6_6, PCL.R2_1
			**8**	aBc: OrG.R6_2, PCun.R4_4; aDc: SFG.R7_7, OrG.R6_2, IPL.R6_3, Hipp.R2_1;<br>aNEg: SFG.R7_7, OrG.R6_2, IPL.R6_3; aNEloc: IFG.R6_1, PrG.R6_6
			**9**	aBc: IFG.R6_1, PhG.R6_5; aDc: IFG.R6_1, OrG.R6_2, IPL.R6_3, Hipp.R2_1;<br>aNEg: SFG.R7_7, OrG.R6_2, IPL.R6_3; aNEloc: STG.R6_1
			**10**	aBc: PCun.R4_4; aDc: SFG.R7_7, OrG.R6_2, IPL.R6_3; aNEg: SFG.R7_7, OrG.R6_2;<br>aNEloc:SFG.R7_4, PCL.R2_1, STG.R6_1

Table 2. Subsets of Predictive Functional Features for RH Group

	CI (%)	Top (%)	Subset	Subsets of Predictive Features
**FC**	**99.7**	**5**	**1-10**	MFG.L7_3-MVOcC.L5_2/5_3; MFG.L7_7-MVOcC.R5_5; IFG.L6_4-MVOcC.R5_3; OrG.L6_1-SPL.L5_1; OrG.L6_2-LOcC.L4_2; OrG.L6_3-IPL.L6_1/PCun.L4_3; MTG.L4_2-BG.L6_5; PhG.L6_3-ITG.L7_1/7_7; IPL.L6_1-BG.L6_1
**TF**	**99**	**20**	**1**	**aBc:** BG.L6_1; **aDc:** FuG.L3_1, PhG.L6_3, BG.L6_1; **aNEg:** FuG.L3_1, PhG.L6_3, BG.L6_1, Tha.L8_8
			**2**	**aBc:** ITG.L7_4, BG.L6_1; **aDc:** FuG.L3_1, PhG.L6_2, PhG.L6_3, BG.L6_1; **aNEg:** FuG.L3_1, PhG.L6_3, BG.L6_1; **aNEloc:** BG.L6_3
			**3**	**aBc:** CG.L7_6, BG.L6_1; **aDc:** PhG.L6_2, PhG.L6_3, BG.L6_1; **aNEg:** FuG.L3_1, PhG.L6_1, BG.L6_1; **aNEloc:** BG.L6_3
			**4**	**aBc:** ITG.L7_4, CG.L7_4, CG.L7_6, BG.L6_1; **aDc:** PhG.L6_2, PhG.L6_3, BG.L6_1; **aNEg:** BG.L6_1; **aNEloc:** BG.L6_3
			**5**	**aBc:** BG.L6_1; **aDc:** FuG.L3_1, PhG.L6_2, PhG.L6_3, BG.L6_1; **aNEg:** FuG.L3_1, PhG.L6_3; **aNEloc:** BG.L6_3
			**6**	**aBc**: ITG.L7_4, BG.L6_1; **aDc:** FuG.L3_1, PhG.L6_3, BG.L6_1; **aNEg:** FuG.L3_1, PhG.L6_3, BG.L6_1, Tha.L8_8; **aNEloc:** BG.L6_3
			**7**	**aBc**: ITG.L7_4; **aDc:** PhG.L6_2, PhG.L6_3, BG.L6_1; **aNEg:** IFG.L6_6, ITG.L7_4, BG.L6_1; **aNEloc:** BG.L6_3
			**8**	**aBc**: ITG.L7_4, CG.L7_6, BG.L6_1; **aDc:** PhG.L6_2, PhG.L6_3, BG.L6_1; **aNEg:** ITG.L7_4, FuG.L3_1, BG.L6_1; **aNEloc:** BG.L6_3
			**9**	**aBc**: ITG.L7_4, BG.L6_1; **aDc:** FuG.L3_1, PhG.L6_2, PhG.L6_3, BG.L6_1; **aNEg:** FuG.L3_1, ITG.L7_4, BG.L6_1
			**10**	**aBc**: ITG.L7_4, CG.L7_1, BG.L6_1; **aDc:** FuG.L3_1, PhG.L6_3, BG.L6_1; **aNEg:** FuG.L3_1, ITG.L7_4, BG.L6_1

Table 3. Performance of MLR Models to Predict Malignancy Degree of Gliomas

	Features	One-side CI (%)	Top (%)	Train Dataset		Validation Dataset	
				AUC (mean±std)	Paired t-test (p value)	AUC (mean±std)	Paired t-test (p value)
LH	FC	99.6	5	0.993±0.005	0.015*	0.861±0.069	0.551
	Age+FC	99.6	5	0.996±0.003		0.853±0.079	
	TF	98	15	0.882±0.017	0.001*	0.734± 0.158	0.098
	Age+TF	98	15	0.896±0.016		0.788±0.150	
RH	FC	99.7	5	1.000±0.000	**1**	1.000±0.000	1
	Age+FC	99.7	5	1.000±0.000		1.000±0.000	
	TF	99.7	5	0.945±0.015	0.001*	0.783±0.015	0.095
	Age+TF	99	20	0.968±0.010		0.897±0.165	

CI: confidence interval for selecting of candidate predictive features; Top: features with top high frequency of selected as candidate predictive features. *: significant intergroup difference of classification performance between MLR model with age as predictive feature and MLR model without age as predictive feature.

Table 4. Regions of Interest in the Brainnetome Atlas

Gyrus	Abbreviation	Label ID.L	Label ID.R	Modified cyto-architectonic
Superior Frontal Gyrus	SFG.L/R7_1	1	2	A8m, medial area 8
	SFG.L/R7_2	3	4	A8dl, dorsolateral area 8
	SFG.L/R7_3	5	6	A9l, lateral area 9
	SFG.L/R7_4	7	8	A6dl, dorsolateral area 6
	SFG.L/R7_5	9	10	A6m, medial area 6
	SFG.L/R7_6	11	12	A9m, medial area 9
	SFG.L/R7_7	13	14	A10m, medial area 10
Middle Frontal Gyrus	MFG.L/R7_1	15	16	A9/46d, dorsal area 9/46
	MFG.L/R7_2	17	18	IFJ, inferior frontal junction
	MFG.L/R7_3	19	20	A46, area 46
	MFG.L/R7_4	21	22	A9/46v, ventral area 9/46
	MFG.L/R7_5	23	24	A8vl, ventrolateral area 8
	MFG.L/R7_6	25	26	A6vl, ventrolateral area 6
	MFG.L/R7_7	27	28	A10l, lateral area 10
Inferior Frontal Gyrus	IFG.L/R6_1	29	30	A44d, dorsal area 44
	IFG.L/R6_2	31	32	IFS, inferior frontal sulcus
	IFG.L/R6_3	33	34	A45c, caudal area 45
	IFG.L/R6_4	35	36	A45r, rostral area 45
	IFG.L/R6_5	37	38	A44op, opercular area 44
	IFG.L/R6_6	39	40	A44v, ventral area 44
Orbital Gyrus	OrG.L/R6_1	41	42	A14m, medial area 14
	OrG.L/R6_2	43	44	A12/47o, orbital area 12/47
	OrG.L/R6_3	45	46	A11l, lateral area 11
	OrG.L/R6_4	47	48	A11m, medial area 11
	OrG.L/R6_5	49	50	A13, area 13
	OrG.L/R6_6	51	52	A12/47l, lateral area 12/47

Gyrus	Abbreviation	Label ID.L	Label ID.R	Modified cyto-architectonic
Precentral Gyrus	PrG.L/R6_1	53	54	A4hf, area 4(head and face region)
	PrG.L/R6_2	55	56	A6cdl, caudal dorsolateral area 6
	PrG.L/R6_3	57	58	A4ul, area 4(upper limb region)
	PrG.L/R6_4	59	60	A4t, area 4(trunk region)
	PrG.L/R6_5	61	62	A4tl, area 4(tongue and larynx region)
	PrG.L/R6_6	63	64	A6cvl, caudal ventrolateral area 6
Paracentral Lobule	PCL.L/R2_1	65	66	A1/2/3ll, area 1/2/3 (lower limb region)
	PCL.L/R2_2	67	68	A4ll, area 4, (lower limb region)
Superior Temporal Gyrus	STG.L/R6_1	69	70	A38m, medial area 38
	STG.L/R6_2	71	72	A41/42, area 41/42
	STG.L/R6_3	73	74	TE1.0 and TE1.2
	STG.L/R6_4	75	76	A22c, caudal area 22
	STG.L/R6_5	77	78	A38l, lateral area 38
	STG.L/R6_6	79	80	A22r, rostral area 22
Middle Temporal Gyrus	MTG.L/R4_1	81	82	A21c, caudal area 21
	MTG.L/R4_2	83	84	A21r, rostral area 21
	MTG.L/R4_3	85	86	A37dl, dorsolateral area 37
	MTG.L/R4_4	87	88	aSTS, anterior superior temporal sulcus
Inferior Temporal Gyrus	ITG.L/R7_1	89	90	A20iv, intermediate ventral area 20
	ITG.L/R7_2	91	92	A37elv, extreme lateroventral area 37
	ITG.L/R7_3	93	94	A20r, rostral area 20
	ITG.L/R7_4	95	96	A20il, intermediate lateral area 20
	ITG.L/R7_5	97	98	A37vl, ventrolateral area 37
	ITG.L/R7_6	99	100	A20cl, caudolateral of area 20
	ITG.L/R7_7	101	102	A20cv, caudoventral of area 20
Fsiform Gyrus	FuG.L/R3_1	103	104	A20rv, rostroventral area 20
	FuG.L/R3_2	105	106	A37mv, medioventral area 37
	FuG.L/R3_3	107	108	A37lv, lateroventral area 37

Gyrus	Abbreviation	Label ID.L	Label ID.R	Modified cyto-architectonic
Parahippocampal	PhG.L/R6_1	109	110	A35/36r, rostral area 35/36
	PhG.L/R6_2	111	112	A35/36c, caudal area 35/36
	PhG.L/R6_3	113	114	TL, area TL (lateral PPHC, posterior parahippocampal gyrus)
	PhG.L/R6_4	115	116	A28/34, area 28/34 (EC, entorhinal cortex)
	PhG.L/R6_5	117	118	TI, area TI(temporal agranular insular cortex)
	PhG.L/R6_6	119	120	TH, area TH (medial PPHC)
Posterior Superior Temporal Sulcus	pSTS.L/R2_1	121	122	rpSTS, rostroposterior superior temporal sulcus
	pSTS.L/R2_2	123	124	cpSTS, caudoposterior superior temporal sulcus
Superior Parietal Lobule	SPL.L/R5_1	125	126	A7r, rostral area 7
	SPL.L/R5_2	127	128	A7c, caudal area 7
	SPL.L/R5_3	129	130	A5l, lateral area 5
	SPL.L/R5_4	131	132	A7pc, postcentral area 7
	SPL.L/R5_5	133	134	A7ip, intraparietal area 7(hIP3)
Inferior Parietal Lobule	IPL.L/R6_1	135	136	A39c, caudal area 39(PGp)
	IPL.L/R6_2	137	138	A39rd, rostrodorsal area 39(Hip3)
	IPL.L/R6_3	139	140	A40rd, rostrodorsal area 40(PFt)
	IPL.L/R6_4	141	142	A40c, caudal area 40(PFm)
	IPL.L/R6_5	143	144	A39rv, rostroventral area 39(PGa)
	IPL.L/R6_6	145	146	A40rv, rostroventral area 40(PFop)
Precuneus	Pcun.L/R4_1	147	148	A7m, medial area 7(PEp)
	Pcun.L/R4_2	149	150	A5m, medial area 5(PEm)
	Pcun.L/R4_3	151	152	dmPOS, dorsomedial parietooccipital sulcus(PEr)
	Pcun.L/R4_4	153	154	A31, area 31 (Lc1)
Postcentral Gyrus	PoG.L/R4_1	155	156	A1/2/3ulhf, area 1/2/3(upper limb, head and face region)
	PoG.L/R4_2	157	158	A1/2/3tonIa, area 1/2/3(tongue and larynx region)
	PoG.L/R4_3	159	160	A2, area 2
	PoG.L/R4_4	161	162	A1/2/3tru, area 1/2/3(trunk region)

Gyrus	Abbreviation	Label ID.L	Label ID.R	Modified cyto-architectonic
Insular Gyrus	INS.L/R6_1	163	164	G, hypergranular insula
	INS.L/R6_2	165	166	vIa, ventral agranular insula
	INS.L/R6_3	167	168	dIa, dorsal agranular insula
	INS.L/R6_4	169	170	vId/vIg, ventral dysgranular and granular insula
	INS.L/R6_5	171	172	dIg, dorsal granular insula
	INS.L/R6_6	173	174	dId, dorsal dysgranular insula
Cingualte Gyrus	CG.L/R7_1	175	176	A23d, dorsal area 23
	CG.L/R7_2	177	178	A24rv, rostroventral area 24
	CG.L/R7_3	179	180	A32p, pregenual area 32
	CG.L/R7_4	181	182	A23v, ventral area 23
	CG.L/R7_5	183	184	A24cd, caudodorsal area 24
	CG.L/R7_6	185	186	A23c, caudal area 24
	CG.L/R7_7	187	188	A32sg, subgenual area 32
MedioVentral Occipital Cortex	MVOcC.L/R5_1	189	190	cLinG, caudal lingual gyrus
	MVOcC.L/R5_2	191	192	rCunG, rostral cuneus gyrus
	MVOcC.L/R5_3	193	194	cCunG, caudal cuneus gyrus
	MVOcC.L/R5_4	195	196	rLinG, rostral lingual gyrus
	MVOcC.L/R5_5	197	198	vmPOS,ventromedial parietooccipital sulcus
Lateral Occipital Cortex	LOcG.L/R4_1	199	200	mOccG, middle occipital gyrus
	LOcG.L/R4_2	201	202	V5/MT+, area V5/MT+
	LOcG.L/R4_3	203	204	OPC, occipital polar cortex
	LOcG.L/R4_4	205	206	iOccG, inferior occipital gyrus
Superior Occipital Gyrus	sOcG.L/R2_1	207	208	msOccG, medial superior occipital gyrus
	sOcG.L/R2_2	209	210	lsOccG, lateral superior occipital gyrus
Amygdala	Amyg.L/R2_1	211	212	mAmyg, medial amygdala
	Amyg.L/R2_2	213	214	lAmyg, lateral amygdala
Hippocampus	Hipp.L/R2_1	215	216	rHipp, rostral hippocampus
	Hipp.L/R2_2	217	218	cHipp, caudal hippocampus

Gyrus	Abbreviation	Label ID.L	Label ID.R	Modified cyto-architectonic
Basal Ganglia	BG.L/R6_1	219	220	vCa, ventral caudate
	BG.L/R6_2	221	222	GP, globus pallidus
	BG.L/R6_3	223	224	NAC, nucleus accumbens
	BG.L/R6_4	225	226	vmPu, ventromedial putamen
	BG.L/R6_5	227	228	dCa, dorsal caudate
	BG.L/R6_6	229	230	dlPu, dorsolateral putamen
Thalamus	Tha.L/R8_1	231	232	mPFtha, medial pre-frontal thalamus
	Tha.L/R8_2	233	234	mPMtha, pre-motor thalamus
	Tha.L/R8_3	235	236	Stha, sensory thalamus
	Tha.L/R8_4	237	238	rTtha, rostral temporal thalamus
	Tha.L/R8_5	239	240	PPtha, posterior parietal thalamus
	Tha.L/R8_6	241	242	Otha, occipital thalamus
	Tha.L/R8_7	243	244	cTtha, caudal temporal thalamus
	Tha.L/R8_8	245	246	lPFtha, lateral pre-frontal thalamus

Reference

Fan L, Li H, Zhuo J, et al. The Human Brainnetome Atlas: A New Brain Atlas Based on Connectional Architecture. Cereb Cortex 2016;26:3508-3526.
